# SCIntRuler: guiding the integration of multiple single-cell RNA-seq datasets with a novel statistical metric

**DOI:** 10.1093/bioinformatics/btae537

**Published:** 2024-09-03

**Authors:** Yue Lyu, Steven H Lin, Hao Wu, Ziyi Li

**Affiliations:** Department of Biostatistics, The University of Texas MD Anderson Cancer Center, Houston, TX 77030, United States; Department of Biostatistics and Data Science, The University of Texas Health Science Center at Houston, Houston, TX 77030, United States; Department of Thoracic Radiation Oncology, Division of Radiation Oncology, The University of Texas MD Anderson Cancer Center, Houston, TX 77030, United States; Faculty of Computer Science and Control Engineering, Shenzhen University of Advanced Technology, Shenzhen, Guangdong 518055, P.R. China; Shenzhen Institute of Advanced Technology, Chinese Academy of Sciences, Shenzhen, Guangdong 518055, P.R. China; Department of Biostatistics, The University of Texas MD Anderson Cancer Center, Houston, TX 77030, United States

## Abstract

**Motivation:**

The growing number of single-cell RNA-seq (scRNA-seq) studies highlights the potential benefits of integrating multiple datasets, such as augmenting sample sizes and enhancing analytical robustness. Inherent diversity and batch discrepancies within samples or across studies continue to pose significant challenges for computational analyses. Questions persist in practice, lacking definitive answers: Should we use a specific integration method or opt for simply merging the datasets during joint analysis? Among all the existing data integration methods, which one is more suitable in specific scenarios?

**Result:**

To fill the gap, we introduce SCIntRuler, a novel statistical metric for guiding the integration of multiple scRNA-seq datasets. SCIntRuler helps researchers make informed decisions regarding the necessity of data integration and the selection of an appropriate integration method. Our simulations and real data applications demonstrate that SCIntRuler streamlines decision-making processes and facilitates the analysis of diverse scRNA-seq datasets under varying contexts, thereby alleviating the complexities associated with the integration of heterogeneous scRNA-seq datasets.

**Availability and implementation:**

The implementation of our method is available on CRAN as an open-source R package with a user-friendly manual available: https://cloud.r-project.org/web/packages/SCIntRuler/index.html

## 1 Introduction

Single-cell RNA sequencing has been widely used in studies of different diseases to characterize cellular heterogeneity and identify phenotype-related cell populations (Van de Sande et al. 2023). For example, scRNA-seq has been used to identify biomarkers that predict treatment responses of complex diseases (Gawel et al. 2019) as well as to assist biomarker discovery for the diagnosis of cancer (Sun et al. 2021). Moreover, the research community has put forth tremendous efforts to establish open-access scRNA-seq consortiums, such as the Human Cell Atlas (HCA) (Regev et al. 2017) and Human Tumor Atlas Network (HTAN) (Rozenblatt-Rosen et al. 2020). Motivated by these public scRNA-seq resources, more researchers seek to incorporate their own study with external data. Including multiple data sources not only leads to an increased sample size, enhancing the robustness of scientific conclusions, but also introduces a variety of biological conditions not covered in individual cohorts. Merging these heterogeneous scRNA-seq data requires appropriate data integration methods to ensure the validity of biological findings. The purpose of data integration methods is to remove platform-specific or subject-specific batch effects. This is critical to prevent masking significant biological signals and harmonize multiple datasets from different experiments, conditions, or samples.

There are a variety of existing integration methods, employing either linear or non-linear techniques and resulting in different versions of batch-corrected data. Overall, the methods can be classified into three major categories. The first class of methods typically uses linear transformations to obtain a low-dimensional representation of the data and align different datasets based on the representations. Seurat Canonical Correlation Analysis (CCA), e.g. uses canonical correlations to find shared patterns in gene expressions and align multiple datasets (Stuart et al. 2019). Graph-based methods use nearest-neighbor graph structures to identify similar cells from different datasets. Harmony, a popular method in this category, projects cells into a shared space that preserves intra-sample relationships while enhancing inter-sample comparability (Korsunsky et al. 2019). The third group is the deep learning-based approach, which uses neural network architectures to model and integrate scRNA-seq data, offering flexibility and the ability to capture non-linear associations. Notable deep learning approaches include scVI (Lopez et al. 2018), trVAE (Lotfollahi et al. 2020), and scGen (Lotfollahi et al. 2019).

Despite the availability of numerous integration methods, it may not be straightforward on how to handle multiple data sources in practice. When two datasets have little batch effects or when the biological signals dominate the difference, simply merging the two data sources is sufficient. Otherwise, unique biological signals may be eliminated after applying integration methods, potentially leading to the loss of important biological variation. When significant batch effects exist, choosing an optimal method becomes the next crucial question. The challenges of batch correction and the potential risk of removing important biological variations have been highlighted in Tyler et al. (2021).

As shown in a comprehensive review of 68 scRNA-seq integration methods by Luecken et al. (2022), there is no single, best integration method. The performance of the methods is dependent on the complexity of the integration task. They reported that CCA performs well on simpler tasks where batch effects are clear and biological variation is less complex. However, CCA tends to prioritize batch effect removal and may over-correct biological signals. Harmony can correct batch effects efficiently and perform well with less complex biological variations. This method; however, is not ranked high for more complex real data tasks with different sources of biological variations. Scanorama tends to perform the best in complex integration tasks where samples span over locations/conditions and contain complex, nested batch effects (Hie et al. 2019), showing robustness in handling both subtle biological variation and strong batch effects. Another systematic review work by Ryu et al. (2023) also finds that there is no single method is superior across all integration scenarios. Therefore, researchers need to carefully select integration methods based on the specific integration task at hand. The real challenge in practice; however, is that the strength of biological similarity between different studies is usually unknown.

To address the various challenges mentioned, we propose a novel statistical metric called *SCIntRuler* to guide the selection of an optimal data integration method. The essence of SCIntRuler lies in its ability to quantitatively assess the similarity of biological signals between different data sources. When the difference between the data sources is dominated by batch effect, i.e. the biological signals are similar across datasets, methods that prioritize batch effect correction can be adopted. When the condition is the opposite, it is recommended to use methods that better preserve biological signals. Moreover, SCIntRuler can effectively identify potentially shared cell subpopulations in heterogeneous scRNA-seq subjects and intuitively visualize the information sharing. Through extensive simulation studies and four different real data applications, we illustrate the utility and favorable performance of the proposed method. We also included the application of three popular data integration methods to verify the choice by SCIntRuler.

## 2 Materials and methods

**Figure 1.**
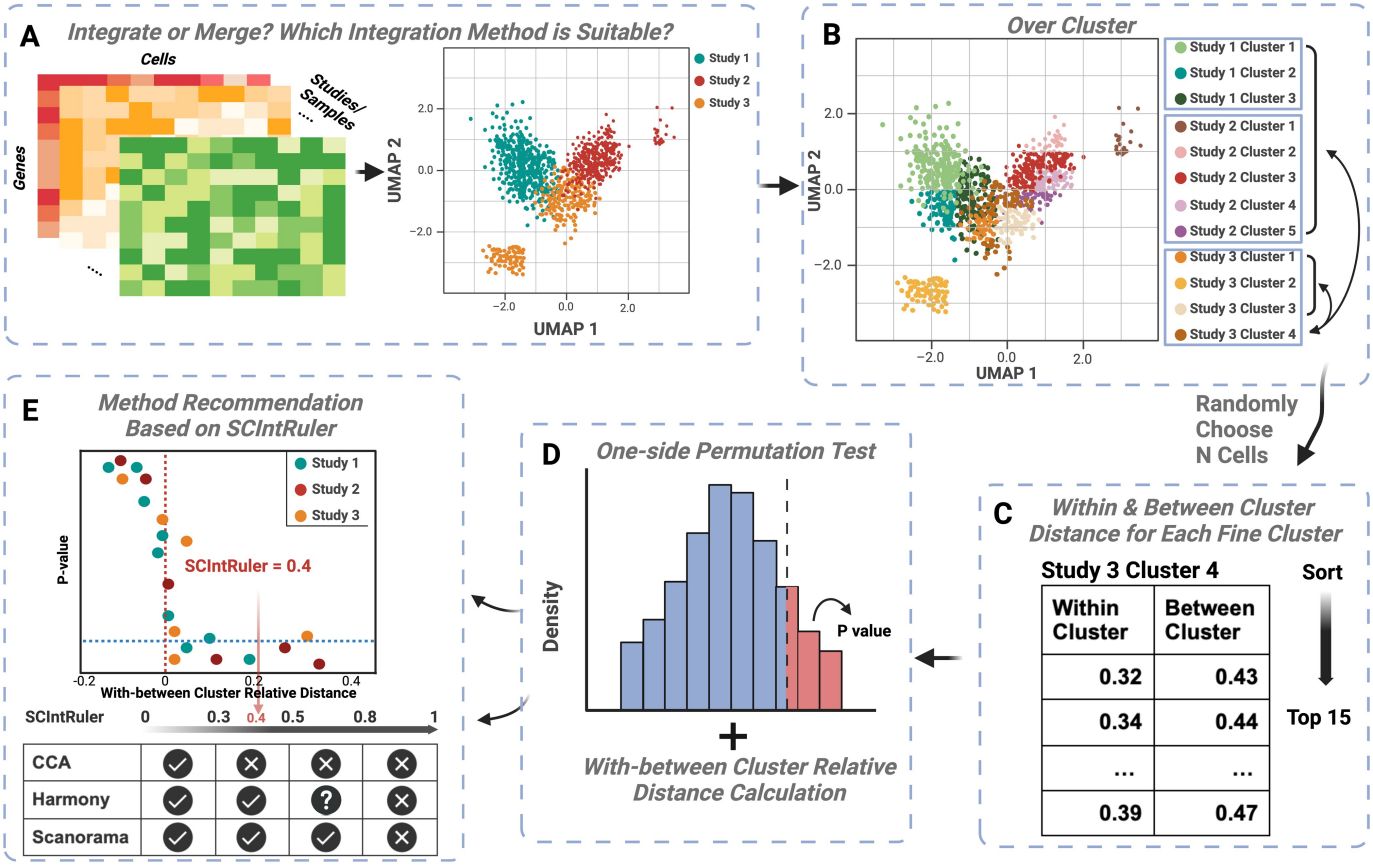
A schematic overview of SCIntRuler workflow. (**A**) The input includes scRNA-seq data and the associated study information. (**B**) Cluster each individual scRNA-seq dataset into broad clusters and fine clusters using the Louvain algorithm. (**C**) Compute within and between (broad) cluster relative distances after randomly selecting fine clusters. (**D**) Perform a one-sided permutation test to examine the null hypothesis that the broad clusters between studies have significant differences if they were measured in one study. (**E**) Visualize the results by drawing a scatter plot with within–between cluster relative distances and permutation *P*-values

The key idea of SCIntRuler is to develop a hypothesis-based testing framework that evaluates within-sample and cross-sample similarities of cell groups. The inputs of SCIntRuler include a scRNA-seq gene expression matrix and the study or batch information. SCIntRuler outputs a numeric ratio that represents the level of information sharing across datasets and a figure illustrating the permutation test-based *P* value versus the relative between–within cluster distances. The figure intuitively demonstrates how the information of the studies is shared and the related statistics. Figure 1 presents the workflow consisting of four steps: (i) fine cluster generation (Fig. 1B), (ii) calculating within–between cluster distance (Fig. 1C), (iii) one-sided permutation testing (Fig. 1D), and (iv) summarization and visualization for method selection (Fig. 1E).

### 2.1 Divide cells into broad and fine clusters

We quantify the similarity of scRNA-seq data sources using the unit of cell clusters. For each data source, we first obtain broad clusters of cells by the Louvain clustering algorithm (Blondel et al. 2008) implemented by Seurat toolkit with resolution = 0.5. To further quantify each broad cluster, we generate fine clusters by implementing the following criteria: If the cell count within a broad cluster is more than 200, the cluster is subdivided randomly into three fine clusters. If the cell count falls within the range of 50 to 200, two fine clusters are generated randomly. Otherwise, the cluster remains unchanged. The purpose of the fine clustering step is to obtain cell groups with smaller sizes within each broad cluster. These fine clusters are used as units to compare within and across data sources to quantify data similarity. As shown in our experiments (see Supplementary Fig. S4), our algorithm can obtain robust results with a wide range of cluster size selections (details in Supplementary Section S3). Because the generation of fine clusters is carried out randomly, each of these fine clusters will be distributed evenly within the broad cluster. The criterion of the fine cluster generation is a parameter that can be modified when users implement SCIntRuler.

### 2.2 Calculate within–between cluster distance

SCIntRuler examines within and between cluster distance matrices, denoted by $D_{\mathrm{within}}$ and $D_{\mathrm{between}}$. The distance matrices are calculated based on cell-cell distance matrices. For a data source $S_{k},k=1,\ldots ,K$, where *K* is the number of data sources, let $X_{ig}$ denote the normalized expression of a gene g=1,…,G in cell $i=1,\ldots ,N_{k}$. G and $N_{k}$ are the number of genes and number of cells, respectively. We use Cosine normalization to remove the impact of sequencing depth (Korsunsky et al. 2019). From the previous step, $N_{k}$ cells are divided into several broad clusters, denoted as $C_{b}^{k},b\in 1,\ldots ,B$ and the fine clusters are denoted as $C_{f}^{k},f\in 1,\ldots ,F$. For each fine cluster, we calculate the Euclidean distance of cells within $C_{f}^{k}$ against all other cells in the same cluster (for $D_{\mathrm{within}}$) and all other cells in other clusters (for $D_{\mathrm{between}}$). As there are usually thousands or even millions of cells, computing the distances for every pair of cells can be computationally infeasible. We randomly select 20 cells from $C_{f}^{k}$ and 500 cells from all other cells of $C_{k}$ in the same data source for the calculation of $D_{\mathrm{within}}$ and 500·(K−1) cells from other data sources for $D_{\mathrm{between}}$.

In summary, each element of within-cluster distance matrix is computed by

$$d_{ij}=\sqrt{\sum_{g=1}^{G}{\left(x_{ig}-x_{jg}\right)}^{2}},\;i\in C_{f}^{k}\;,\;j\in S_{k}\;,\;j\notin C_{f}^{k}\;\;\;$$

and each element of between-cluster distance matrix is

$$d_{ih}=\sqrt{\sum_{g=1}^{G}{\left(x_{ig}-x_{hg}\right)}^{2}},\;i\in C_{f}^{k}\;,\;h\notin S_{k}$$

### 2.3 One-sided permutation test

This step is to assess if within-cluster distance is significantly smaller than between-cluster distance. A significant result indicates that the cells are much closer to the cells from the same source as compared to the other sources, suggesting little shared information across different datasets. For each of the 20 randomly selected cells, we identify the 15 nearest neighbors among the 500 cells from the same data source ($d_{\mathrm{within}}$). This results in a total of 300 distance measurements, which populate the $d_{\mathrm{within}}$ array. Similarly, we identify the 15 nearest neighbors among the 500·(K−1) cells from other data sources ($d_{\mathrm{between}}$). This also results in a total of 300 distance measurements, which populate the $d_{\mathrm{between}}$ array. Both $d_{\mathrm{within}}$ and $d_{\mathrm{between}}$ arrays consist of 300 elements, representing the smallest distances for the selected cells.

We then performed a permutation test to compare the two arrays. We choose to use the permutation test as it does not require stringent distribution assumptions of the distances. To determine whether the within-cluster cells are closer or not, we quantify the difference of $d_{\mathrm{within}}$ and $d_{\mathrm{between}}$ by $T={\bar{d}}_{\mathrm{between}}-{\bar{d}}_{\mathrm{within}}$. Then null and alternative hypotheses are $H_{0}:\mu_{\mathrm{between}}\leq \mu_{\mathrm{within}};\;H_{0}:\mu_{\mathrm{between}}>\mu_{\mathrm{within}}$. We run *N* permutations to form a null distribution of the test statistic, which can be approximated by its asymptotic distribution, which is computed using a randomized quasi-Monte Carlo method (Genz and Bretz 2009). The *P* value $P_{H0}(T\geq T_{\mathrm{obs}})$ is computed by $\#\{\hat{T}\geq {\hat{T}}_{\mathrm{obs}}\}/N$. The stability of the SCIntRuler metric to the choice of k-nearest neighbor and different *P*-value thresholds was evaluated in Supplementary Sections S5 and S6.

### 2.4 SCIntRuler metric

The magnitude of within- and between-cluster distance reflects the similarity level of the different data sources. When within-cluster distance is much smaller than between-cluster distance, it suggests that the data sources are quite heterogeneous. We develop a novel metric to quantify the extent of this difference, called relative within–between distance (RWBD). The structure of RWBD is similar to a silhouette score (Rousseeuw 1987), defined as

$$\mathrm{RWBD}=({\bar{d}}_{\mathrm{between}}-{\bar{d}}_{\mathrm{within}})/\max({\bar{d}}_{\mathrm{between}},{\bar{d}}_{\mathrm{within}})$$

RWBD lies in the range of −1 to 1. As RWBD approaches 1, the relative distance between clusters compared to the distance within clusters increases, indicating a clear distinction among the integrated data sources.

**Figure 2.**
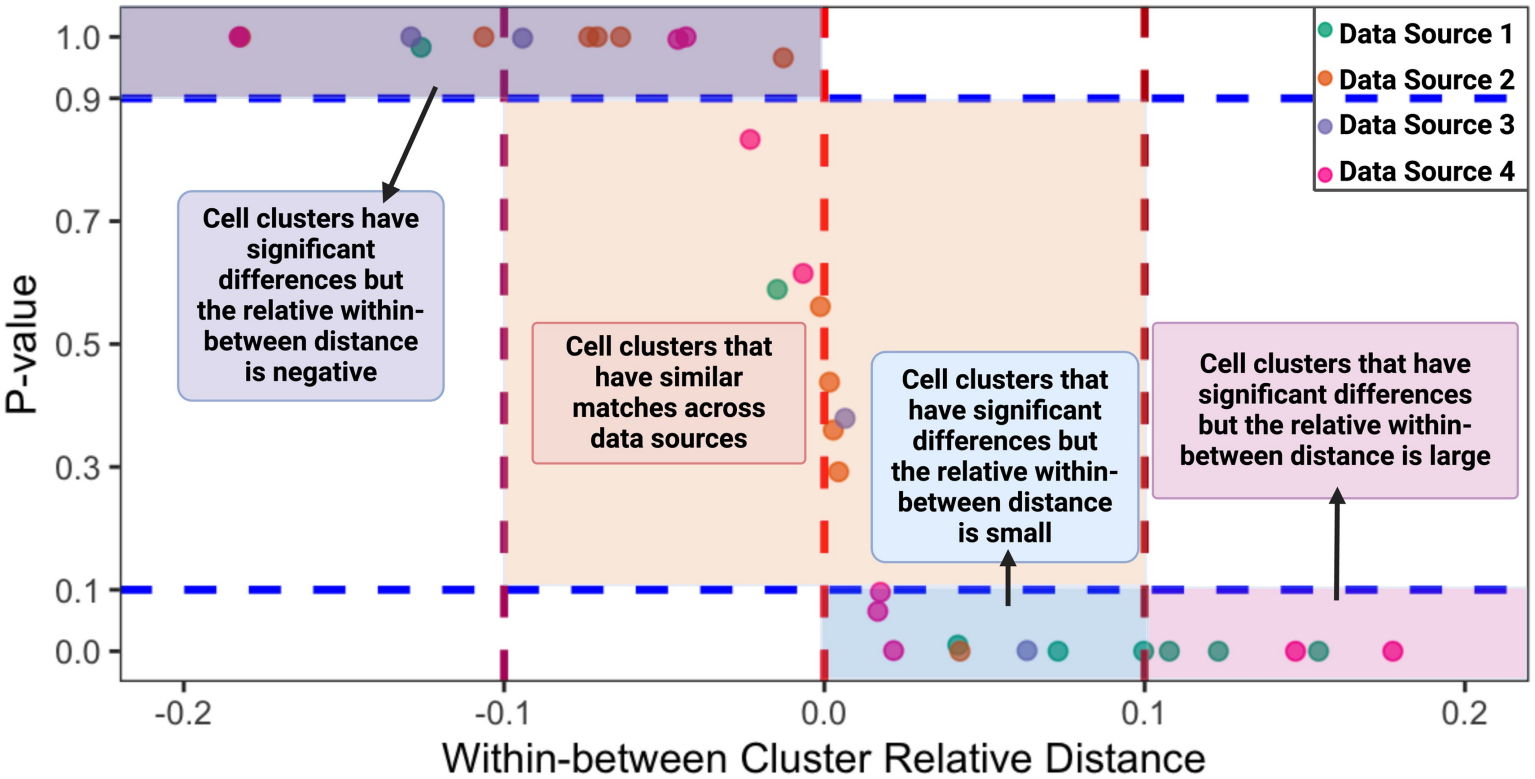
SCIntRuler plot illustration. SCIntRuler plot interpretation, where each point represents a fine cell cluster. The *P*-value against the within–between cluster relative distance for all the clusters are plotted, categorized by data sources

To marry the information from both RWBD and permutation test results, we define SCIntRuler score as the proportion of fine clusters with *P*-values below .1 and a positive RWBD by comparing the within-cluster and between-cluster distances for fine clusters. The SCIntRuler score falls between 0 and 1, quantifying how well-separated the clusters are. The closer the SCIntRuler score is to 1, the more distinct the subjects are from each other, implying that the batch effects are considerable and that a sophisticated integration method would be beneficial to resolve these effects and better harmonize the data. This metric, alongside a user-friendly R package on Github, provides researchers with a direct measure of SCIntRuler and an associated visualization. We illustrated the meaning of a SCIntRuler plot in Fig. 2.

**Figure 3.**
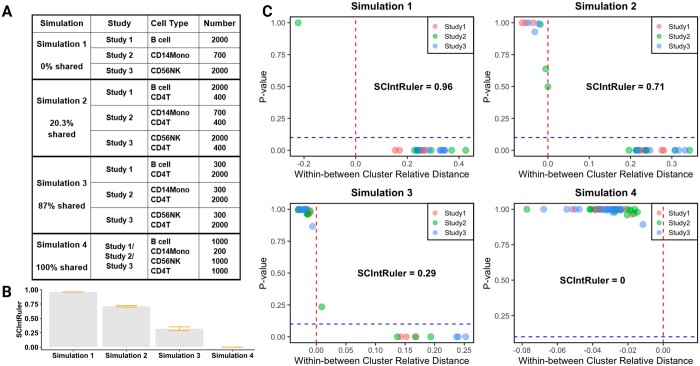
Simulation settings and results. (**A**) Simulation settings with cell type number information. (**B**) Bar plot with error bars showing the mean and three times standard deviation of SCIntRuler based on 50 Monte Carlo datasets under each simulation setting. (**C**) Visualization of SCIntRuler results for simulation settings 1, 2, 3, and 4

## 3 Result

### 3.1 Simulation results

We conducted simulation studies to evaluate SCIntRuler’s efficacy of guiding integration selection across scenarios with varying shared information levels among datasets, using simulation data from a real PBMC (Peripheral Blood Mononuclear Cells) scRNA-seq dataset. Simulations depicted scenarios from no shared signal (three distinct cell types) to full overlap (100% shared cell types), progressively increasing CD4 T helper cell numbers across three data sources to mimic increasing information sharing(Details of simulation setting were in Supplementary Section S2).

Figure 3C visualizes the SCIntRuler scores for each simulation. We can observe a clear trend that the score decreases with an increase in shared information among the datasets. The result indicates that our algorithm has robust SCIntRuler score estimations in each setting with small standard errors. 50 Monte Carlo iterations under each simulation scenario are summarized as bar plots. We also showed the within–between cluster relative distance versus *P* value by SCIntRuler in Fig. 3C based on one simulation dataset in each simulation. Simulation 1 with least shared information was displayed with the highest SCIntRuler score(close to 1). The distinct and separate cell populations across studies imply that a simple merge of the datasets is adequate, given the minimal overlap and clear delineation of cell types. In Simulation 2, with a SCIntRuler score of 0.71, there is a noticeable but not large overlap of cell types across the datasets, showing a moderate level of shared information. The integration is essential to adjust for these effects and align the shared cell populations, ensuring that the integrated dataset accurately reflects the biological composition. Thus, the methods that offer a balance between correcting for batch effects and maintaining biological variation are best. For Simulation 3, the SCIntRuler score decreases further to 0.29, reflecting a high degree of shared information among the cell populations across studies. This suggests that while the cell types are largely similar, subtle data source-specific effects may still exist. In this scenario, data integration methods that prioritize the preservation of biological signals should be preferred to protect subtle signals and ensure overall data integrity. Finally, in Simulation 4, which yields a SCIntRuler score of 0, the data are fully shared across studies. This would typically suggest a need for integration. In such cases of complete homogeneity, any integration method would perform well, as the cell types are inherently well-aligned.

To further illustrate which integration method is more suitable under different settings, we visualize the data without integration (simply merging the single cell objects) and after applying three popular data integration methods in Supplementary Fig. S1. The UMAP (Uniform Manifold Approximation and Projection) visualizations across the simulations indicate that the choice of integration method significantly impacts the resulting data integration. CCA tends to over-integrate in scenarios with lower shared information, as the cell groups from different cell identities are forced to merge together in the first two panels from the second column of Supplementary Fig. S1. In contrast, Harmony offers a middle ground, which presents reasonable integration in simulations 1 and 2, but forces the merging of the red and green cell groups in simulation 3. Scanorama shows the capacity to retain the underlying data structure (Supplementary Fig. S1C, last column), suggesting its suitability in both low- and high-shared information scenarios. These results indicate that different data integration methods should be applied in different settings, and SCIntRuler can provide guidance for the selection of an effective method. Across various scenarios, from no shared information to completely shared datasets, SCIntRuler accurately reflected the level of data complexity and the corresponding need for integration.

### 3.2 Real data

We also investigated the use of SCIntRuler to guide the selection of a data integration method in four real data applications. We considered four scenarios, human brain datasets (Yang et al. 2022), human breast cancer only (Wu et al. 2021), mixed cancer [breast cancer and liver cancer (Ma et al. 2020)], and primary myelofibrosis (CD34+) (Parenti et al. 2021), each containing data from multiple heterogeneous sources to merge/integrate. Based on the biological nature of these studies, these data range from relatively homogeneous to relatively heterogeneous. We used them to illustrate the practicality of SCIntRuler in identifying the need for and choice of data integration. All real datasets were downloaded from GEO (Barrett et al. 2012) and loaded into the Seurat v5.0.1 (Hao et al. 2023) for data filtration, normalization, scale, dimension reduction, and visualization (details in Supplementary Section S1).

#### 3.2.1 Application to human brain data

**Figure 4.**
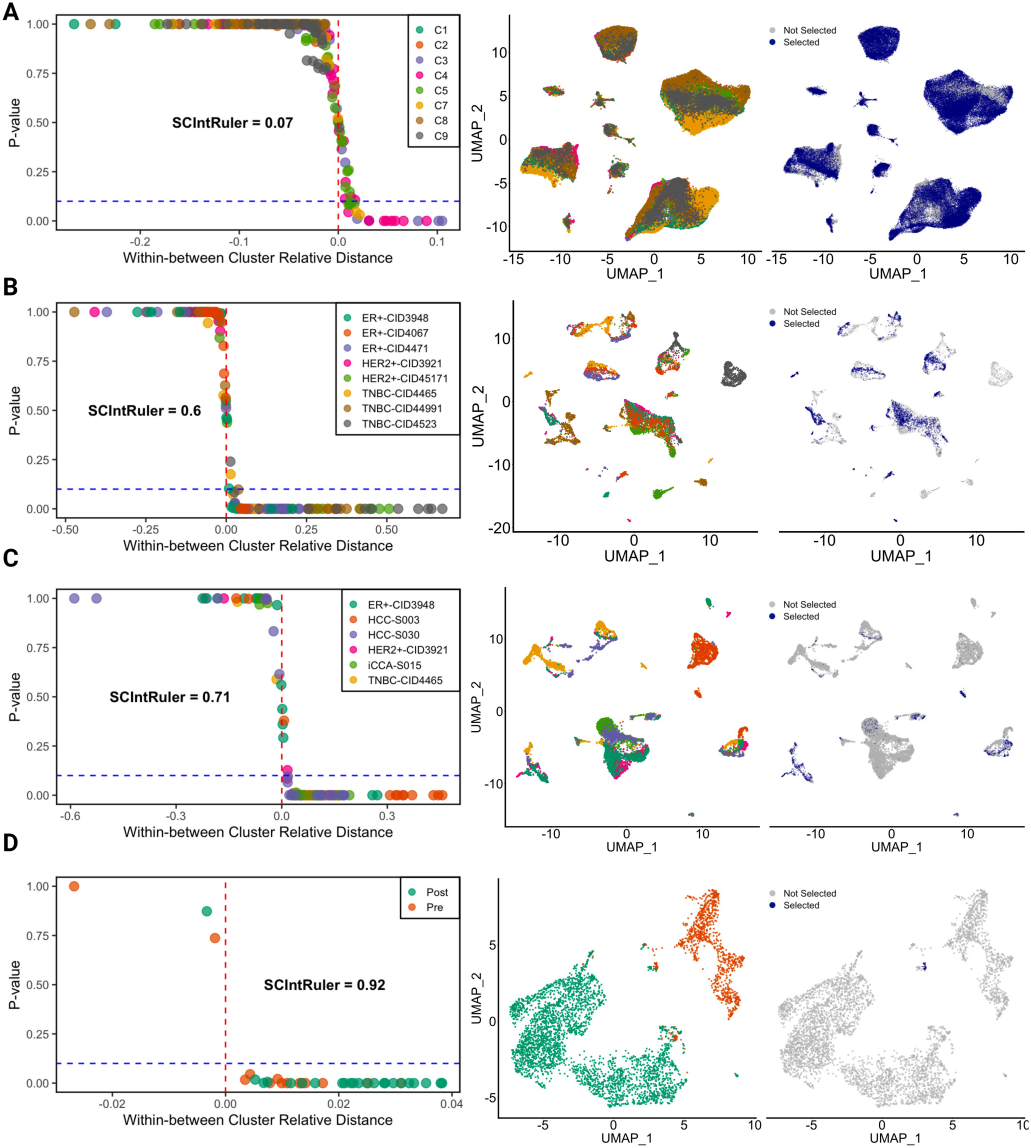
Results for real data application. Results of applying SCIntRuler to various real datasets: human brain (**A**), breast cancer (**B**), mixed cancer types (**C**), and primary myelofibrosis pre- and post-treatment (**D**). The left panel shows SCIntRuler scores and different colors represent different clusters or cell types as identified in each dataset. The right panel marks cells in fine clusters with significant between-group differences (*P*-values > .9, negative relative distances), indicating the cells share cell type identity across conditions. Selected cells within fine clusters are marked by solid markers, and non-selected cells are represented by empty markers

The first scRNA-seq dataset was obtained from the normal brain tissue of eight individuals. This dataset was originally explored to study the contribution of vascular cells to Alzheimer’s disease. The dataset contains single-nucleus transcriptomes from 25 hippocampus and cortex samples, including nine individuals diagnosed with Alzheimer’s disease and eight cognitively normal controls, aged between 58 and 93 years. For our analysis, we focused on the samples from the eight normal controls to obtain a relatively homogeneous dataset. As visualized in Fig. 4A, SCIntRuler yields a score of 0.07. This low score aligns with the observed large *P*-values, suggesting that the inherent similarity between different subjects is high. As a result, complex integration is not required here. The right panel visualized in Fig. 4 of the UMAP plot demonstrates a high degree of overlap with a large proportion of “Selected” cells. From Supplementary Fig. S3A, any integration method would perform well for such datasets.

#### 3.2.2 Application to human cancer data

##### 3.2.2.1 Breast cancer only

In our analysis, we leveraged a subset of the comprehensive breast cancer dataset detailed in the referenced study (Ma et al. 2020). Specifically, we used scRNA-seq data from eight patients, carefully selecting samples that encompassed the three primary breast cancer subtypes to capture a broad spectrum of the disease. We included three samples from the estrogen receptor-positive (ER+) group, two from the human epidermal growth factor receptor 2 positive (HER2+) group, and three from the triple-negative breast cancer (TNBC) group, ensuring each had a cell count within the range of 1000–2000. This selection criteria aimed to provide a balanced representation of the tumor microenvironment across different breast cancer subtypes for our integrative analysis. In Fig. 4B, both the SCIntRuler score, which rises to 0.6, and the “selected” cells in the UMAP visualization indicate that the different data sources (i.e. patients) contain moderate homogeneity. A higher score suggests a larger distinction between cell populations, which requires careful selection of integration methods to ensure that biological variation is accurately represented. Scanorama performs better in biological variations conservation compared to Harmony and CCA (Supplementary Fig. S3B).

##### 3.2.2.2 Mixed cancer types

In this real data analysis, we merged breast cancer data with liver cancer data from a study that included 37 liver cancer patients, representing both hepatocellular carcinoma (HCC) and intrahepatic cholangiocarcinoma (iCCA). From this cohort, two HCC samples (H72, H58a) and one iCCA sample (C60) were selected based on their availability of tumor biopsies and the diversity of patient conditions, such as age and viral hepatitis status. These were integrated with three chosen samples from the breast cancer dataset, each representing a different subtype: ER+, HER2+, and TNBC, to explore the cellular heterogeneity and molecular complexity across different cancer types. Compared to the “Breast cancer only” data, this dataset has more heterogeneity among patients. The SCIntRuler score of 0.71 is consistent with greater distinctions between the two cancer types, as compared with just one type of cancer. The UMAP plot in the right panel of Fig. 4 showcases a smaller proportion of “selected” cells, which also exhibit more distinct clustering patterns and less shared information. We observe that both Scanorama and Harmony perform well by preserving biological variations in this situation (Supplementary Fig. S3C). CCA may over-correct the biological signal in this application.

#### 3.2.3 Application to primary myelofibrosis data

The last dataset includes pre- and post-treatment scRNA-seq data of CD34+ cells from a patient with primary myelofibrosis. The SCIntRuler score of 0.92 reflects intense changes at the cellular level of pre- and post-treatment, presenting massive heterogeneity in treatment response. This significant shift reflects the treatment’s influence on bone marrow cell populations and clonal evolution in primary myelofibrosis, potentially leading to changes in gene expression and progression toward more aggressive conditions like acute myeloid leukemia (Parenti et al. 2021). The high SCIntRuler score reflects that there is no need to integrate, as visualized by the disparate cell clusters and barely shared information in the UMAP plot. CCA and Harmony succeed in removing batch effects but fail to conserve biological variation (Supplementary Fig. S3D).

The application of SCIntRuler to real scRNA-seq datasets from various biological conditions has highlighted its robustness and versatility in guiding data integration decisions.

## 4 Discussion

**Figure 5.**
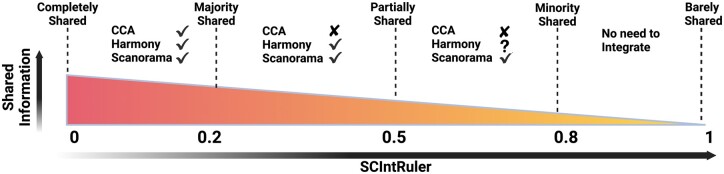
SCIntRuler chart for scRNA-seq data integration method selection. The SCIntRuler value inversely correlates with the amount of shared information between datasets. The less shared information there is between multiple datasets, the larger the SCIntRuler score

We developed a novel statistical method, SCIntRuler, which visualizes the information-sharing level across different data sources and provides quantitative measurement to guide the selection of a suited data integration method. SCIntRuler exhibited robust performance in simulations and real-data analyses across diverse dataset complexities, establishing its reliability for selecting integration methods in various data scenarios. It effectively measures the shared information among scRNA-seq datasets and demonstrates subtle differences in data heterogeneity. This helps protect biological variations and avoids unnecessary integration. We developed a data integration recommendation chart in Fig. 5, which depicts the suitability of various integration methods like CCA, Harmony, and Scanorama across different data-sharing scenarios. For SCIntRuler values below .2, the multiple data sources share a majority of the information. Methods like CCA, Harmony, and Scanorama are all suitable to remove batch effects and integrate data. When the SCIntRuler score is around 0.5, the multiple data sources share part of the information. We recommend Harmony and Scanorama in this scenario, as they balance batch correction with biological variation conservation. When SCIntRuler reports a score greater than 0.8, we recommend simply merging the data sources for downstream analysis, as little information is shared between the sources, and the differences are driven by biological signals.

We are not the first work to guide the selection of scRNA-seq analysis methods. Existing works that have a similar spirit include Dong et al. (2024), which provides a framework for selecting an optimal method to perform scRNA-seq trajectory analysis. The SCIntRuler algorithm does not rely on any cell type information, allowing for its early application in the analysis pipeline when cell annotations are not yet available. Meanwhile, our exploration of the cell type labels demonstrated high consistency between matched fine clusters across data sources (Supplementary Fig. S7). This finding underscores the biological consistency using our distance-based matching.

A few parameters are involved in the SCIntRuler method, such as the clustering precision, thresholds of defining broad and fine clusters, number of representative cells to compute distance, etc. Although these factors play a role in the computation, we performed extensive simulation experiments to evaluate the sensitivity of the model against different parameter selections. Supplementary Sections 3, 4, 5, and 6 describe our sensitivity analysis and show that SCIntRuler consistently yields reliable guidance for method selection across a range of parameter settings. Our implementation also allows users to manually specify the parameters when needed. We also provide additional details regarding the selection of score thresholds in the recommendation chart (details in Supplementary Section S9).

We evaluated the computational performance of SCIntRuler on both simulated and real scRNA-seq datasets, with varying numbers of cells and genes. Our empirical results demonstrate that the computational time scales with both the number of cells and genes. Despite the variability in hardware specifications, software configurations, system load, and data characteristics, our method is designed to be scalable and can handle these variations effectively (details in Supplementary Table S1).

One limitation of the current framework is that we only demonstrated three popular data integration methods in the experiment: CCA, Harmony, and Scanorama. We acknowledge that the primary focus of this work is to illustrate SCIntRuler and not to comprehensively evaluate existing data integration methods. The review works by Argelaguet et al. (2021), Luecken et al. (2022), and Ryu et al. (2023) systematically compared more scRNA-seq data integration methods, such as scGen (Lotfollahi et al. 2019), LIGER (Liu et al. 2020), FastMNN (Zhang et al. 2019), and scVI (Lopez et al. 2018). Each integration method is designed with unique strengths and thus may offer optimal solutions for scenarios not covered by the three methods we assessed. Nonetheless, different methods can have similar performance and thus fit easily to the guidance chart by the SCIntRuler guidance chart. For example, scGen uses cell-type information to improve integration, and it tends to prioritize biological variation conservation. It performs well in tasks similar to those handled by Scanorama. LIGER, just like Harmony, shows a similar balance between batch correction and conservation of biological variation. FastMNN, like Scanorama, also uses mutual nearest neighbors to find anchors between batches, tending to perform well in integrating complex datasets. A detailed discussion on the concordance between our experimental methods and other existing methods can be found in Supplementary Section S8. This variety in integration methods again highlights the complexity of scRNA-seq data integration. SCIntRuler provides a valuable starting point for method selection, and researchers are encouraged to consider broader methods for their data. Future work extending SCIntRuler’s applicability by assessing these additional methods in different data scenarios would provide more exhaustive validation and guidance.

## Supplementary Material

btae537_Supplementary_Data

## Data Availability

Human brain data can be downloaded from: https://www.ncbi.nlm.nih.gov/geo/query/acc.cgi?acc=GSE163577. Breast cancer data can be downloaded from: https://www.ncbi.nlm.nih.gov/geo/query/acc.cgi?acc=GSE176078. Liver cancer data can be downloaded from: https://www.ncbi.nlm.nih.gov/geo/query/acc.cgi?acc=GSE151530. Primary myelofibrosis data can be downloaded from: https://www.ncbi.nlm.nih.gov/geo/query/acc.cgi?acc=GSE176078.
