# Supplementary material for: SCIntRuler: guiding the integration of multiple single-cell RNA-seq datasets with a novel statistical metric
Source: Bioinformatics. 2024 Sep 3;40(9):btae537. doi: 10.1093/bioinformatics/btae537 (PMC11415828; doi:10.1093/bioinformatics/btae537)
Supplement: btae537_Supplementary_Data [file btae537_supplementary_data.zip › 7.pdf]

Supplementary materials for “SCIntRuler: Guiding the integration of multiple single-cell RNA-seq datasets with a novel statistical metric”

Yue Lyu[1,2], Steven H. Lin[3], Hao Wu[4,5], Ziyi Li[1,*]

1. Department of Biostatistics, The University of Texas MD Anderson Cancer Center, Houston, TX 77030, USA.
2. Department of Biostatistics and Data Science, The University of Texas Health Science Center at Houston, Houston, TX 77030, USA.
3. Department of Thoracic Radiation Oncology, Division of Radiation Oncology, The University of Texas MD Anderson Cancer Center, Houston, TX 77030, USA.
4. Faculty of Computer Science and Control Engineering, Shenzhen University of Advanced Technology, Shenzhen, 518055, P.R. China
5. Shenzhen Institute of Advanced Technology, Chinese Academy of Sciences, Shenzhen, 518055, P.R. China

All correspondence should be addressed to Ziyi Li (zli16@mdanderson.org).

ORCID for Yue Lyu: 0000-0002-8912-6624, Hao Wu: 0000-0003-1269-7354, Steven H. Lin: 0000-0003-4411-0634, Ziyi Li: 0000-0001-8359-0533

S1. Pre-processing workflow with Seurat

We used them to illustrate the practicality of SCIntRuler in identifying the need for and choice of data integration. All real datasets were download from GEO[1] and loaded into the Seurat v5.0.1[2] within the R (version 4.3.1) for data flirtation, normalization, scale, dimension reduction and visualization. This section details the standardized pipeline adopted for the initial handling of real datasets. This pre-processing strategy is pivotal to ensuring the quality and reliability of downstream analyses[3]. The UMI count matrix was loaded and quality control criteria was implemented: cells are expected to have a minimum of 500 UMIs and at least 250 genes detected to ensure adequate sequencing depth and complexity. The mitochondrial gene expression ratio is also considered, with a threshold set to exclude cells with a ratio higher than 5% to eliminate dying cells or those with mitochondrial contamination. Gene expression was also filtered to include only those genes present in 10 or more cells. These carefully selected thresholds serve to maintain the biological integrity of the data while ensuring the removal of low-quality cells.

S2. Simulation setting

We conducted a series of simulation studies to assess the efficacy of SCIntRuler in guiding the integration selection under different scenarios with varying degrees of shared information among datasets. We generated the simulation data based on a real peripheral blood mononuclear cell (PBMC) scRNA-seq dataset. In each study, we randomly drew different numbers of CD4 T helper cells, B cells, CD14 monocytes, and CD56 NK cells to mimic four real-world scenarios with three data sources (Fig 3A). In the first scenario, we simulated

a situation when three different data sources contained completely different cell types and thus no shared biological signal across the data sources. In total, we randomly drew 4,700 cells from B cell, CD14 Mono cell, and CD56 NK cell; each cell type represented one data source. In the second to fourth scenarios, we gradually included increasing numbers of CD4 T helper cells in the three data sources. This mimicked the level of information sharing increase in these simulation settings. Simulation 2 introduced a moderate overlap, with 20.3% cells sharing the same cell type identity, while simulation 3 presented a higher similarity with 87% shared cell types. Simulation 4 represented an extreme case where all cell types were shared, i.e., 100% similarity.

S3. Evaluating Criteria for Fine Cluster division

In our supplementary analysis, we rigorously assessed the criteria used for fine cluster division in simulation datasets. Specifically, we examined the impact of varying the cell count thresholds for subdividing broad clusters. For clusters exceeding 100 cells, the subdivision into three fine clusters was contrasted with the established 200-cell threshold, while for those surpassing 20 cells, a comparison was made with the standard 50-cell threshold for dividing into two fine clusters. Our simulations demonstrated that the choice between the (20,100) and (50,200) thresholds yielded consistent SCIntRuler scores(Figure S4A), underscoring the robustness of our methodology. This consistency was observed across all simulation datasets, suggesting that our fine clustering approach is resilient to variations in cell count thresholds and can be reliably used in diverse analytical contexts.

S4. Robustness to Different Cell Selection Criteria

The robustness of the SCIntRuler methodology against varying cell selection criteria was evaluated to ensure the reliability of our cluster analysis under different parameters. While the default analysis employed the selection of 20 cells from fine clusters and 500 cells from other clusters, we extended our analysis to include two additional sets of parameters: (20, 200) and (50, 1000). We conducted 50 iterations for each parameter set under each simulation scenario, applying the default parameters otherwise. The SCIntRuler scores across different simulations remained stable(Figure S5), indicating that our method is resilient to changes in cell selection criteria. This stability in scores confirms the SCIntRuler's applicability and accuracy across various analytical conditions.

S5. Robustness to choice of k nearest neighbors

The robustness of the SCIntRuler methodology against the selection of k nearest neighbors was evaluated to ensure the reliability of our cluster analysis under varying parameters. Previously employing the top 15 nearest neighbors for distance calculations, we extended our analysis to include a range from the top 1 to the top 15 nearest neighbors. We ran 50 times for each k under each simulation scenario by applying default parameters. The SCIntRuler scores across different simulations remained remarkably stable, indicating that our method is resilient to changes in the number of nearest neighbors considered (Figure S6). This stability in scores confirms the SCIntRuler's applicability and accuracy.

S6. Robustness to choice of p-value of permutation test

The stability of the SCIntRuler metric was further established by evaluating its sensitivity to different p-value thresholds used in the permutation test. Our initial pipeline utilized a p-value threshold of 0.1; however, to test the robustness of our approach, we also employed a more stringent threshold of 0.05. We ran 50 times for both p = 0.05 and p = 0.1 under each simulation scenario.The resulting SCIntRuler scores were remarkably similar(Figure S4B), indicating that our metric is not overly sensitive to the choice of p-value threshold. This robustness ensures that the SCIntRuler can be applied with confidence across a range of statistical level.

S7. Illustration of SCIntRuler plot

In this section, we present Figure 2, which offers a comprehensive analysis of the SCIntRuler's functionality and its application in data integration.

Figure 2 interprets the SCIntRuler plot, showing how each point, representing a cell cluster's p-value in relation to its within-between cluster distance, varies across different data sources. Data points with negative relative distances to the left indicate significant internal cluster differences, revealing distinct cell profiles. In contrast, points near the center suggest minimal distance variations, implying cellular profile similarities across datasets. Points to the right with positive relative distances highlight the heterogeneity within clusters.

Figure 5 acts as a reference for selecting suitable data integration methods according to SCIntRuler scores. This part of the figure aligns the application of Seurat CCA[4], Harmony[5], and Scanorama[6] with the degree of shared information within the datasets. The recommendation scale spans from a full endorsement of integration methods for significantly shared information to the advice against integration when shared data is minimal.

S8. Extension of SCIntRuler with Additional Integration Methods

The primary focus of our work is to illustrate SCIntRuler's utility rather than to comprehensively evaluate all existing data integration methods. While our study focused on demonstrating SCIntRuler's effectiveness using the three most commonly used integration method Seurat CCA, Harmony, and Scanorama, SCIntRuler is designed to be flexible. We acknowledge that there are numerous integration methods available, each with unique strengths suited for different scenarios. Review papers by Luecken et al. (2021) systematically compare various scRNA-seq data integration methods, such as LIGER, FastMNN, and scVI, among others. Our framework provides a robust starting point that can be extended to these methods.

For example, LIGER, on the other hand, prioritizes the removal of batch effects over the conservation of biological variation. While effective at integrating datasets, especially across species and cell types, LIGER tends to retain broad cell-type variation but may remove nuanced biological differences. LIGER's strong batch removal capabilities, similar to Harmony, allow it to fully merge batches within cell-type clusters, although it may sometimes leave batch structures within specific cell types.

FastMNN also demonstrates strong performance by balancing the removal of batch effects with the preservation of biological variation. It performs well in conserving trajectory structure and utilizes mutual nearest neighbors to find anchors between batches, similar to Scanorama. FastMNN's approach to dimensionality reduction, using PCA or singular value decomposition, combined with MNN techniques, makes it a promising method for complex integration tasks, particularly for scATAC-seq data.

scVI, along with other deep learning-based methods like trVAE and scGen, is particularly adept at modeling non-linear associations. Similar to Scanorama, scVI has shown outstanding performance on complex real-world datasets, demonstrating robustness in preserving complex biological variations and strong batch effects. Additionally, methods like scVI and Scanorama are adept at managing strong batch effects from single cells while preserving spatial and rare cell type variations. Among the deep learning methods, scVI consistently shows top performance across various tasks and data types, making it a versatile and reliable choice for complex scRNA-seq data integration.

To further validate SCIntRuler's utility, we worked on additional simulations using other integration methods such as LIGER, FastMNN and scVI (Figure S3). Future work will extend SCIntRuler's applicability by assessing these additional methods in various data scenarios, further enhancing its utility for the research community.

S9. Rationale behind the score thresholds in the recommendation chart

The score thresholds in SCIntRuler's recommendation chart were primarily established based on observations from our simulation studies and real data applications. In each experiment, we recorded the SCIntRuler scores and evaluated which integration method performed best under different scenarios. **For Simulation 1 - 4:**

Simulation 1: Completely Separate Cell Types

SCIntRuler Score: 0.95

Rationale: In this simulation, the datasets consisted of entirely distinct cell types with no overlap. The high SCIntRuler score indicated minimal shared biological information between the datasets. When applying various integration methods, we found that simple merging of the datasets without additional integration was sufficient and preserved the biological distinctions. Methods like Seurat CCA, which aggressively integrates different datasets, could obscure these biological differences and is less suitable for this scenario. We set the threshold for recommending no integration at scores above 0.8, as datasets with such high scores generally represent cases where biological signals are distinct and integration might obscure meaningful differences.

Simulation 2: Moderate Overlap Between Datasets

SCIntRuler Score: 0.70

Rationale: This simulation depicted datasets with some overlap in cell types, leading to a moderate to high SCIntRuler score. Here, integration was necessary to adjust for batch effects and align the shared cell populations. Scanorama and scVI were particularly effective in this scenario. Scanorama managed to correct the batch effects while retaining the complex structure of the cell types, and scVI, as a deep learning approach, was good at handling non-linear relationships and preserving subtle biological variations. The SCIntRuler score in this range (0.5-0.8) indicated that while the datasets shared information, there were still some distinctions that needed to be preserved.

Simulation 3: High Overlap with Some Batch Effects

SCIntRuler Score: 0.30

Rationale: This simulation featured datasets with a high degree of overlap in cell types but with noticeable batch effects. The lower SCIntRuler score indicated that most of the biological information was shared across datasets. Here, methods like Scanorama and Harmony were effective in integrating the datasets and removing batch effects. Harmony balanced batch correction with the conservation of biological variation. Based on this, we set the threshold for recommending Scanorama, Harmony, and similar methods with score between

0.2 and 0.5, where integration is more beneficial to harmonize datasets without losing important biological information.

Simulation 4: Highly Similar Datasets with No Significant Batch Effects

SCIntRuler Score: 0.00

Rationale: In this simulation, the data were fully shared across studies, yielding an SCIntRuler score of 0. This score indicated complete homogeneity among the studies. As study-specific biological signals are little and the cell types are inherently well-aligned, any integration method would perform well. This scenario reinforced our decision to recommend integration methods for datasets with SCIntRuler scores close to 0 (below 0.2).

For real data analysis:

Primary Myelofibrosis Dataset: Extreme Heterogeneity

SCIntRuler Score: 0.92

Rationale: The dataset from a patient with primary myelofibrosis, including pre- and post-treatment samples, presented a scenario of extreme heterogeneity due to the significant cellular changes following treatment. The high SCIntRuler score of 0.92 indicated that integration was not required, as the datasets displayed very distinct cell populations with minimal shared information. This was further supported by the UMAP plot, which showed disparate cell clusters with little overlaps (Supplementary Figure S3 Panel D). This scenario contributed to our decision to set the threshold for recommending no integration around scores above 0.8, where datasets are highly divergent.

Mixed Cancer Types Dataset: High Heterogeneity

SCIntRuler Score: 0.71

Rationale: This dataset combined breast cancer and liver cancer samples, representing a more heterogeneous scenario compared to the breast cancer dataset alone. The SCIntRuler score of 0.71 reflected the greater distinctions between the two cancer types. In such cases, integration methods that balance batch correction with the preservation of biological variation, like Scanorama, was effective (Supplementary Figure S3 Panel C). The higher SCIntRuler score indicated that more careful integration was needed to handle the increased differences, supporting the recommendation to use these methods for datasets with moderate to high variability.

Breast Cancer Dataset: Moderate Heterogeneity

SCIntRuler Score: 0.60

Rationale: In this dataset, we analyzed scRNA-seq data from eight breast cancer patients, representing three primary breast cancer subtypes. The SCIntRuler score of 0.60 indicated a moderate level of shared information, reflecting the variability among different breast cancer subtypes. In this context, an effective integration method was necessary to capture the biological complexity while preserving the unique characteristics of each subtype. Scanorama was identified as the most suitable method for this scenario, as it performed well in maintaining biological variation compared to Harmony and CCA (Supplementary Figure S3 Panel B). This led us to set the threshold for recommending integration methods like Scanorama in range 0.5 to 0.8, where datasets share information but retain some distinct biological signals.

Human Brain Dataset: High Homogeneity

SCIntRuler Score: 0.07

Rationale: The human brain dataset, which consists of samples from eight cognitively normal individuals, was selected to represent a relatively homogeneous scenario. The low SCIntRuler score of 0.07 indicated that the inherent similarity between different subjects was high. This finding is consistent with the observation that any integration method would perform adequately for such homogeneous datasets (Supplementary Figure S3 Panel A). The score threshold applies to scenarios like this, where SCIntRuler values below 0.2

suggest that any integration method would be appropriate due to the high similarity across datasets.

While these thresholds were based on our observations, we recognize that they are somewhat arbitrary and may not be optimal for all data scenarios. The ideal cutoffs may vary depending on the specific integration context and algorithms used. Further refinement of these thresholds will require applying SCIntRuler across a wider range of datasets and integration scenarios. The chosen thresholds represent the best options from our experiments and serve as a useful starting point. We plan to refine these cutoffs in future work as more data and feedback become available.

References

[1] Tanya Barrett, Stephen E Wilhite, Pierre Ledoux, Carlos Evangelista, Irene F Kim, Maxim Tomashevsky, Kimberly A Marshall, Katherine H Phillippy, Patti M Sherman, Michelle Holko, et al. Ncbi geo: archive for functional genomics data sets—update. Nucleic acids research, 41(D1):D991–D995, 2012.

[2] Yuhan Hao, Tim Stuart, Madeline H Kowalski, Saket Choudhary, Paul Hoffman, Austin Hartman, Avi Srivastava, Gesmira Molla, Shaista Madad, Carlos Fernandez-Granda, and Rahul Satija. Dictionary learning for integrative, multimodal and scalable single-cell analysis. Nature Biotechnology, 2023.

[3] Mary Piper, Meeta Mistry, Jihe Liu, William Gammerdinger, and Radhika Khetani. scrna-seq lessons from hcbc. Zenodo, Jan 2022.

[4] Tim Stuart, Andrew Butler, Paul Hoffman, Christoph Hafemeister, Efthymia Papalexi, William M Mauck, Yuhan Hao, Marlon Stoeckius, Peter Smibert, and Rahul Satija. Comprehensive integration of single-cell data. Cell, 177(7):1888–1902, 2019.

[5] Vincent D Blondel, Jean-Loup Guillaume, Renaud Lambiotte, and Etienne Lefebvre. Fast unfolding of communities in large networks. Journal of statistical mechanics: theory and experiment, 2008(10):P10008, 2008.

[6] Brian Hie, Bryan Bryson, and Bonnie Berger. Efficient integration of heterogeneous single-cell transcriptomes using scanorama. Nature biotechnology, 37(6):685–691, 2019.

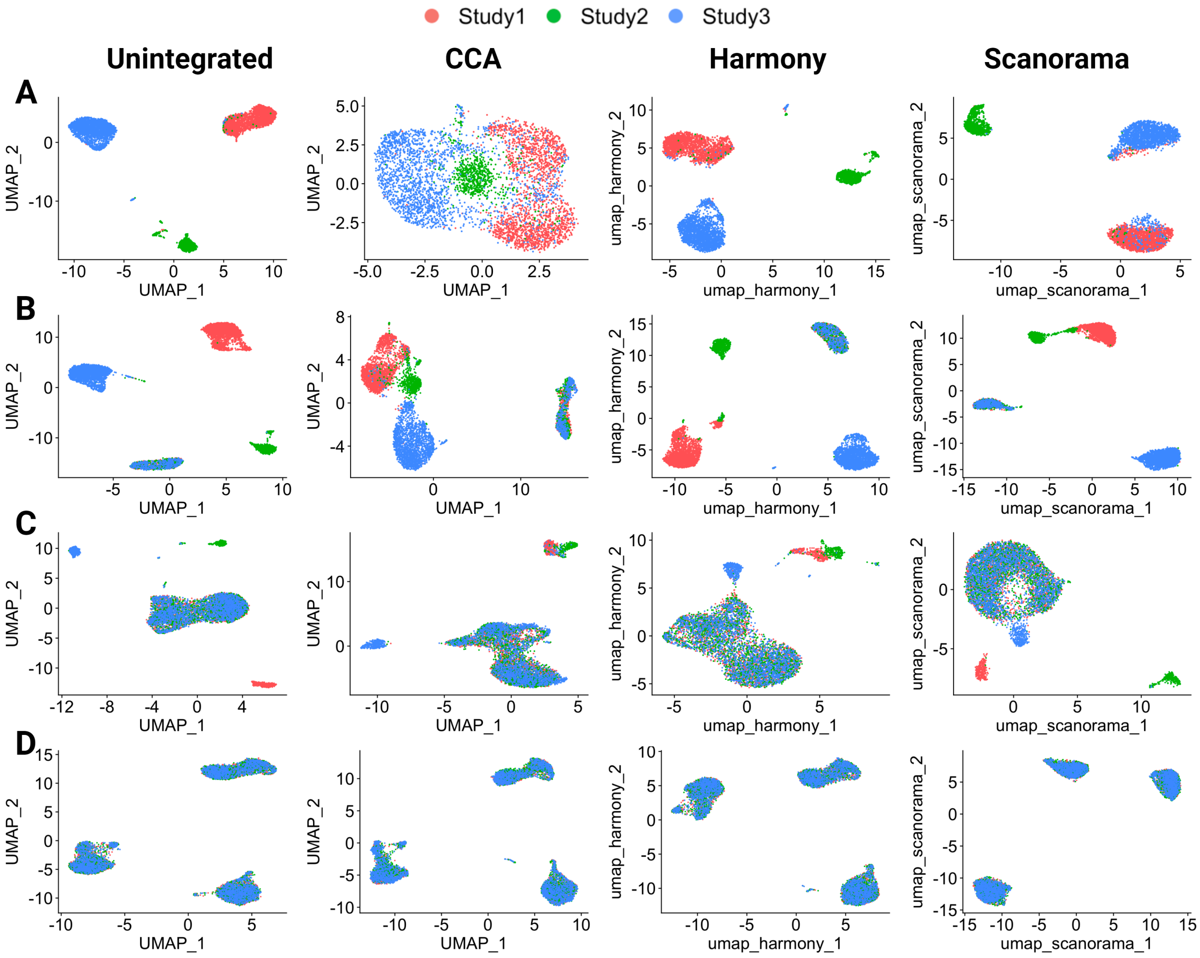

Figure S1: **Results for different simulation settings using popular data integration methods.** Visualization of unintegrated data and results after applying the integration methods (CCA, Harmony, Scanorama). Panels A, B, C, and D correspond to simulation settings 1, 2, 3, and 4, respectively.

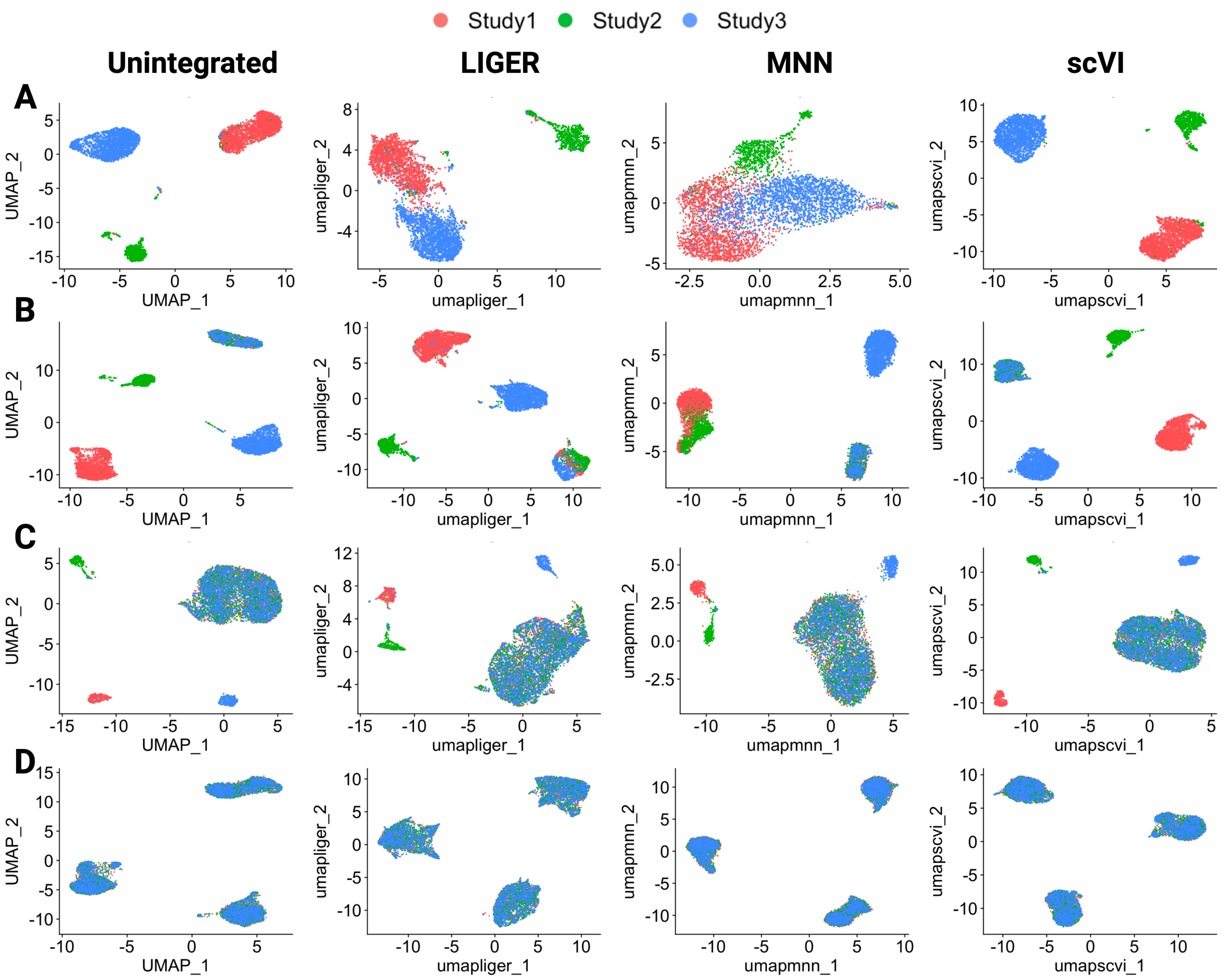

Figure S2: **Results for different simulation settings using other data integration methods.** Visualization of unintegrated data and results after applying the integration methods(LIGER, FastMNN, scVI). Panels A, B, C, and D correspond to simulation settings 1, 2, 3, and 4, respectively.

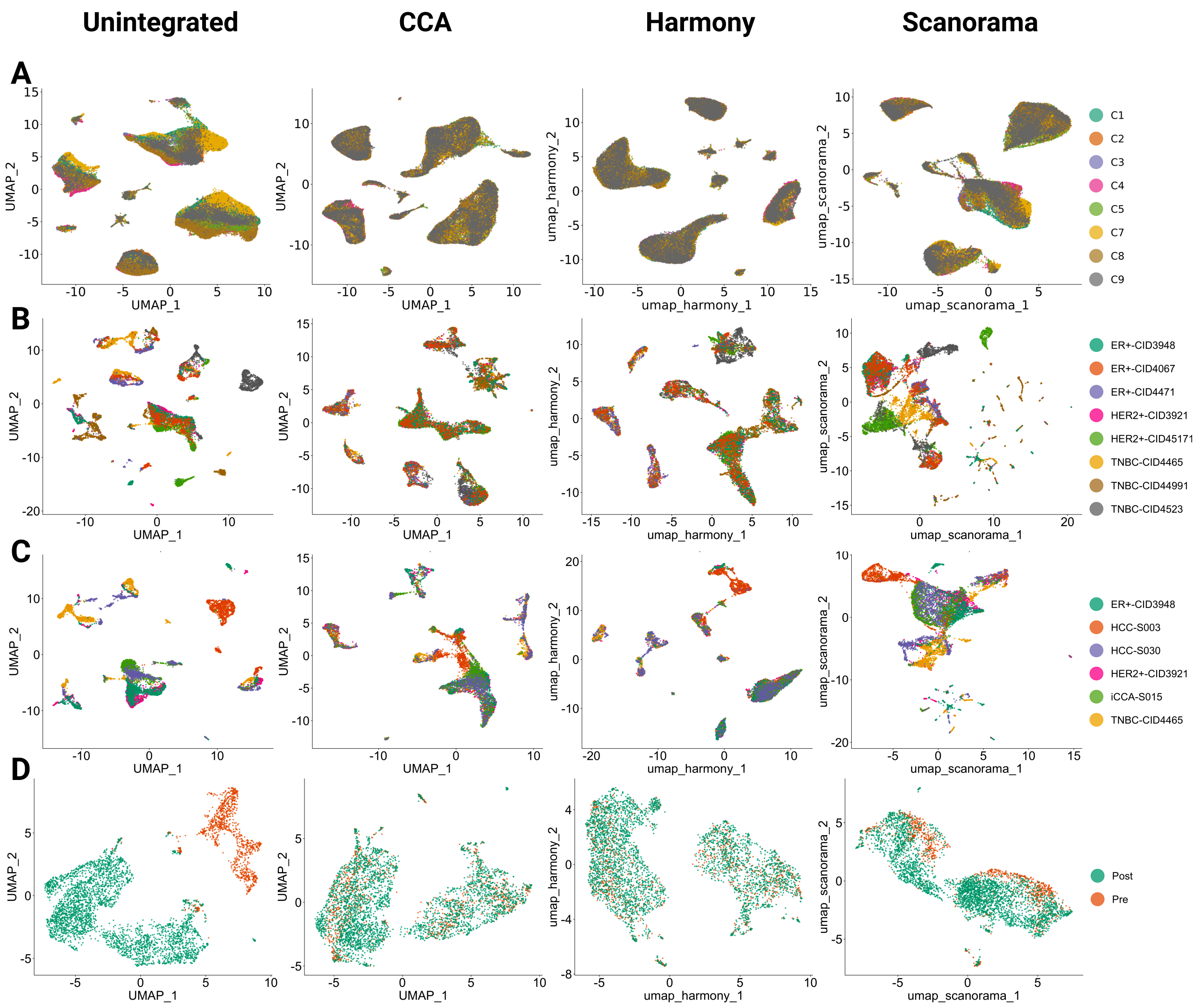

Figure S3: **Results for real datasets using popular data integration methods.** Visualization of unintegrated data and results after applying the integration methods(CCA, Harmony, Scanorama). Panels A, B, C, and D correspond to brain data, breast cancer, mixed cancer, and primary myelofibrosis data respectively.

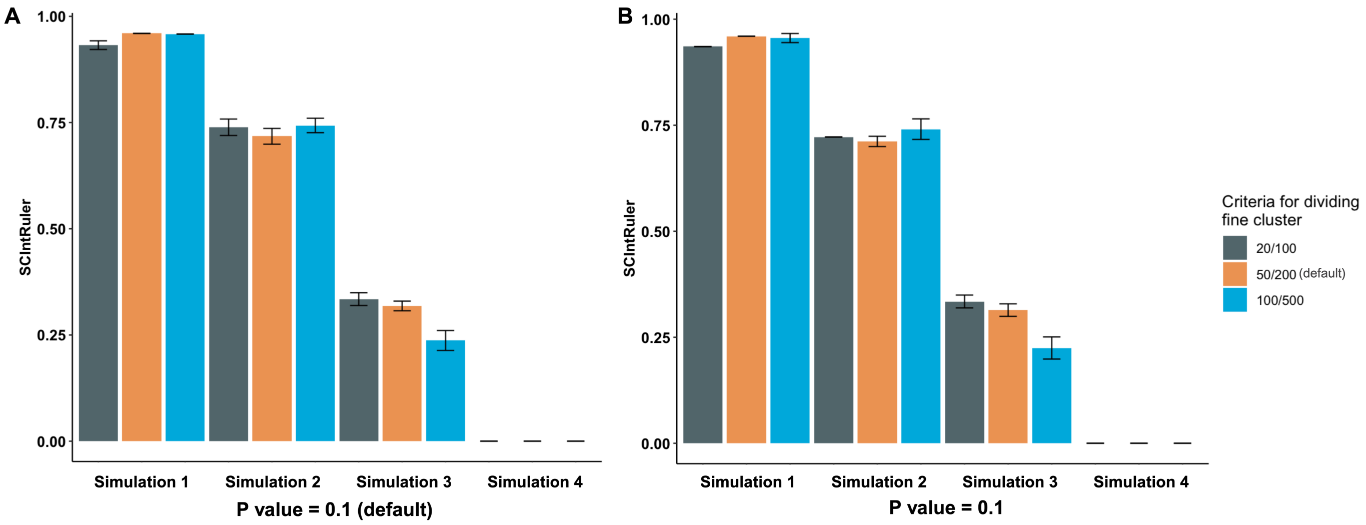

Figure S4: **Robustness of SCIntRuler to Different Fine Clustering Criteria and P-value Thresholds.** Bar plot with error bars showing the mean and standard deviation of SCIntRuler based on 50 Monte Carlo datasets under each criteria setting.

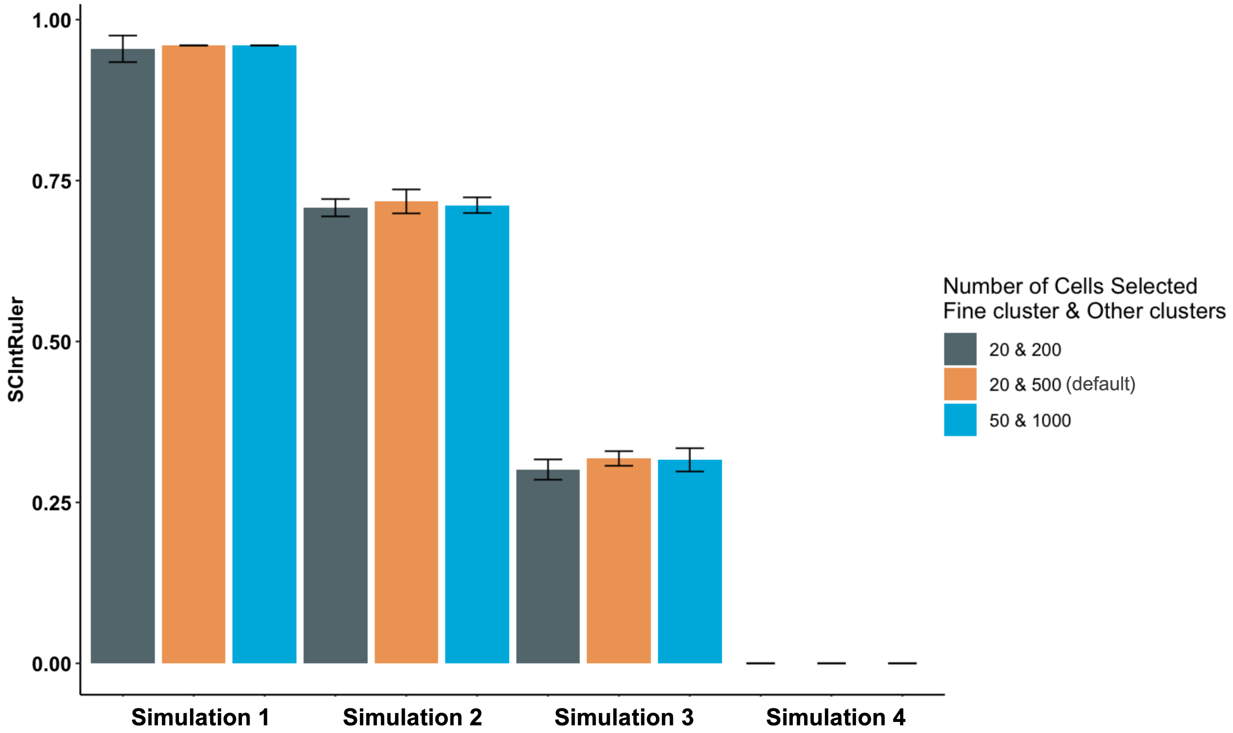

Figure S5: **Robustness of SCIntRuler Methodology to Different Cell Selection Criteria.** The plot illustrates the mean SCIntRuler scores with error bars representing the standard deviation, derived from 50 Monte Carlo simulations for each cell selection criterion. Four distinct simulations were assessed, demonstrating the SCIntRuler method's robustness and consistency across a range of cell selection parameters.

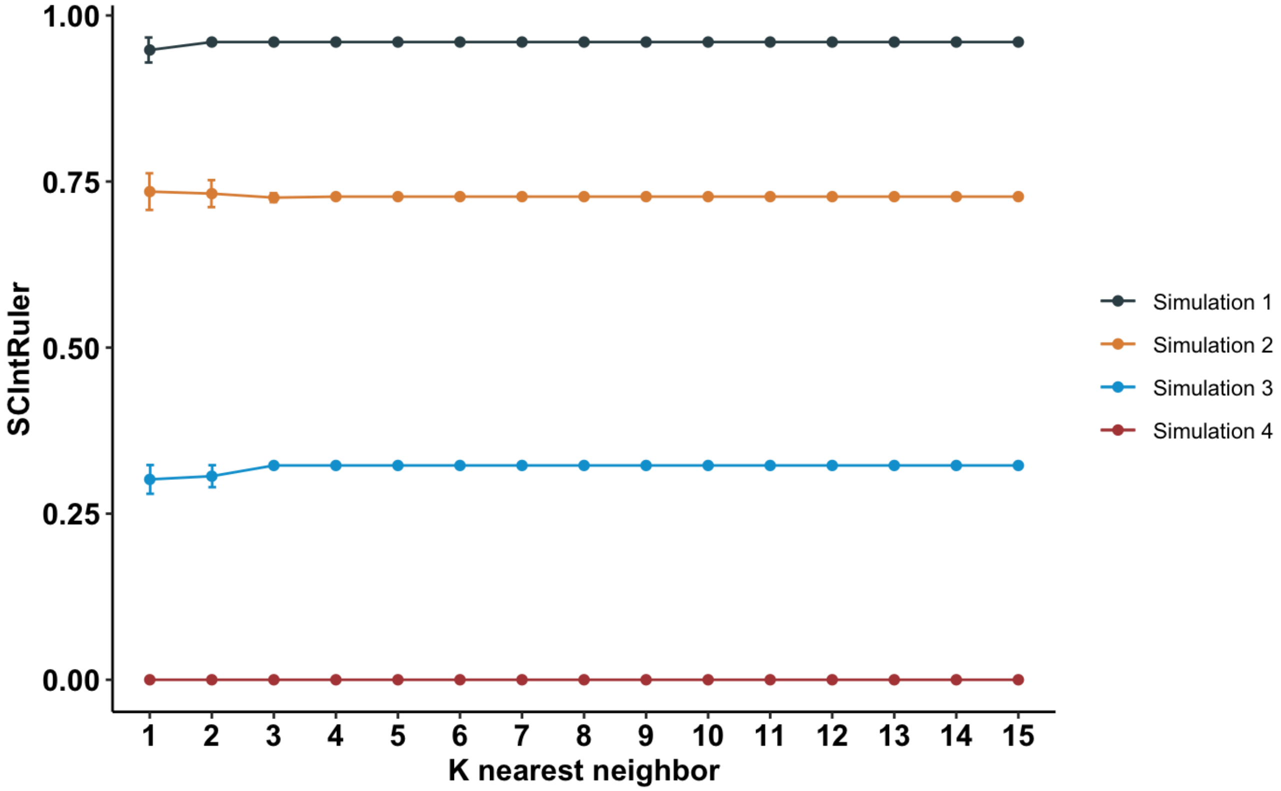

Figure S6: **Robustness of SCIntRuler Methodology to Selection of K Nearest Neighbors.** The plot illustrates the mean SCIntRuler scores with error bars representing the standard deviation, derived from 50 Monte Carlo simulations for each k nearest neighbor setting. Four distinct simulations were assessed, demonstrating the SCIntRuler method's robustness and consistency across a range of k values.

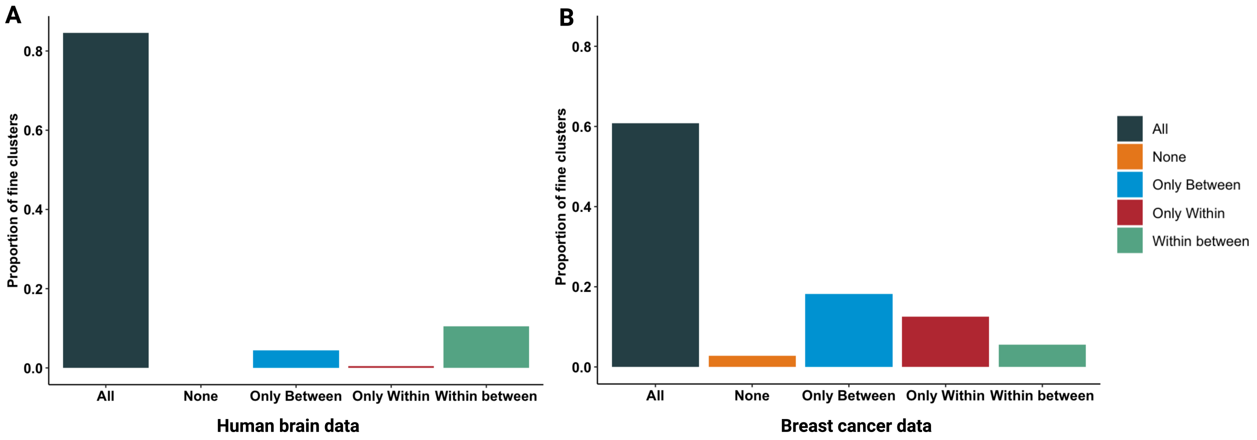

Figure S7: **Cell type consistency for fine clusters matched by SCIntRuler in brain and breast cancer data.** Panel A: results using the brain data. The bars represent the proportion of fine clusters based on cell type consistency categories. "All" indicates that the cell type within the fine cluster matches the cell type of the selected within-group and between-group cell subgroups. "Only Between" and "Only Within" denote fine clusters where the cell type matches exclusively with between-group or within-group cell subgroups, respectively. "Within between" indicates cases where the cell type does not match within the fine cluster but does match when comparing the selected within-group and between-group cell subgroups. "None" suggests no consistency in cell type across any of the groups, indicating heterogeneity. Panel B: results using the breast cancer data. The bar chart follows the same categorization as Panel A, displaying the distribution of cell type consistency within fine clusters for breast cancer data. Both analysis only include fine clusters where a predominant cell type comprises over 90% of the cells. Both panels aim to demonstrate the cell type distribution within fine clusters after applying SCIntRuler, emphasizing the method's ability to identify biologically meaningful sub-clusters during distance-based matching process.

Table S1. Computational Time for simulation and real datasets

Dataset	Number of subjects	Number of genes	Number of Cells	Computational Time
Simulation 1	3	~ 30,000	~ 5,000	~ 35s
Simulation 2	3	~ 30,000	~ 6,000	~ 41s
Simulation 3	3	~ 30,000	~ 7,000	~ 45s
Simulation 4	3	~ 30,000	~ 9,000	~ 50s
Human Brain	8	~ 22,000	~ 70,000	~ 2,500s
Breast Cancer	8	~ 22,000	~ 11,000	~ 500s
Mixed Cancer	6	~ 23,000	~ 8,000	~ 600s
Primary Myelofibrosis	2	~ 15,000	~ 7,000	~ 38s
